# New reference genomes of honey bee-associated bacteria *Paenibacillus melissococcoides, Paenibacillus dendritiformis,* and *Paenibacillus thiaminolyticus*

**DOI:** 10.1128/MRA.00209-23

**Published:** 2023-08-02

**Authors:** Benjamin Dainat, Simone Oberhaensli, Florine Ory, Vincent Dietemann

**Affiliations:** 1 Swiss Bee Research Center, Agroscope Liebefeld, Bern, Switzerland; 2 Interfaculty Bioinformatics Unit and SIB Swiss Institute of Bioinformatics, University of Bern, Bern, Switzerland; 3 Department of Ecology and Evolution, Biophore, UNIL-Sorge, University of Lausanne, Lausanne, Switzerland; Indiana University, Bloomington, Bloomington, Indiana, USA

**Keywords:** brood pathogen, European foulbrood, honey bee health, *Paenibacillus dendritiformis*, *Paenibacillus melissococcoides*, *Paenibacillus thiaminolyticus*

## Abstract

We sequenced the genomes of recently discovered *Paenibacillus melissococcoides* (CCOS 2000) and of the type strains of closely related *P. thiaminolyticus* (DSM 7262) and *P. dendritiformis* (LMG 21716). The three genomes set the basis to unambiguous diagnostic of these honey bee associated *Paenibacillus* bacteria.

## ANNOUNCEMENT

Several bacteria species of the *Paenibacillus* genus are associated with the honey bee, *Apis mellifera*. Among them, *P. larvae* (1) causes American foulbrood, a highly contagious disease, which impedes colony development and can lead to its death. *P. melissococcoides* (2) and *P. dendritiformis* (3) were isolated from colonies affected by European foulbrood (4). *P. thiaminolyticus* was isolated from hive material (5, 6). Because of their close 16S rRNA genetic relatedness (2), *P. melissococcoides* could have been wrongly identified as *P. dendritiformis* or *P. thiaminolyticus* in previous studies.

Here, we present the genome sequences of *P. melissococcoides* CCOS 2000 and of the type strains *P. dendritiformis* LMG 21716 and *P. thiaminolyticus* DSM 7262. The latter bacteria were obtained from BCCM and DSMZ culture collections, respectively. *P. melissococcoides* was found in worker jelly droplets cultured on EFB Basal medium (7, 8) under anaerobic conditions for 4 days at 36°C. The sampled colony was located near Reutigen (46° 41′ 39″ N, 7° 37′ 13″ E), Switzerland.

The three bacteria were grown in 10 mL liquid Basal medium at 36°C overnight. High-molecular-weight genomic DNA was recovered using the GES method of DNA extraction (9) and assessed for quantity, quality, and purity using a Qubit 4.0 fluorometer (dsDNA HS Assay kit; Q32851, Thermo Fisher Scientific, Waltham, MA, USA), an Advanced Analytical FEMTO Pulse instrument (Genomic DNA 165 kb Kit; FP-1002-0275, Agilent, Santa Clara, CA, USA), and a Denovix DS-11 UV-Vis spectrophotometer. Multiplexed SMRTbell libraries were prepared according to PacBio guidelines, Part Number 101-696-100 Version 06 (March 2020). One microgram of gDNA in 100 µL was sheared in a g-TUBE (Covaris, Woburn, MA, USA), concentrated, and cleaned using AMPure PB beads. Samples were quantified and qualified to be in the range of 12–15 kb using the Qubit and the FEMTO instruments, respectively. Libraries were pooled using the PacBio microbial multiplexing calculator. Prior to and after size selection, the library pool was purified using AMPure PB beads. Size selection was performed with a BluePippin instrument (BLU0001; Sage Science, Beverly, MA, USA) using BluePippin with dye free, 0.75% Agarose Cassettes, and S1 Marker (Sage Science; BLF7510) wherein the selection cut-off was set at 6,000 bp. Library pool concentration and size was again assessed using the Qubit and FEMTO instruments, respectively. The final library pools were on average 11.4 kb in size.

PacBio Sequencing primer v4 and Sequel DNA Polymerase 3.0 were annealed and bound, respectively, to the DNA template libraries. Libraries and Spike-In internal control were diffusion loaded at an on-plate concentration of 10 or 11 pM. Sequencing was performed in continuous long read (CLR) mode on the Sequel System with Sequencing kit 3.0, SMRTCells 1M v3, and a 2 h pre-extension followed by 600 min movie time.

CLRs were assembled with the microbial assembly pipeline (https://www.pacb.com/products-and-services/analytical-software/smrt-analysis/, SMRTlink v9.0.0.92188) with default parameter settings (except microasm_coverage = 25 and microasm_genome_size = 6.8 Mb). The pipeline does automatic overlap identification, trimming, and rotation of circular sequences.

Raw data and assembly statistics are summarized in Table 1.

**TABLE 1:** Sequencing statistics and genome data availability of *Paenibacillus melissococcoides*, *Paenibacillus dendritiformis, and Paenibacillus thiaminolyticus*^*a*^

	Data for:		
Parameter	*P. melissococcoides*	*P. dendritiformis*	*P. thiaminolyticus*
No. of polymerase reads, N50	29,360, 71,000	24,865, 70,800	26,390, 50,900
No. of subreads, N50	157,996, 7,100	154,543, 6,700	107,539, 6,900
No. of contigs	21, two of them circular	1, linear	4, one circular
N50 (bp)	594,566	n.a.^*b*^	6,594,752
L50	4	n.a.	1
Assembly size (bases)	7,186,093	6,722,799	6,609,466
Contig type, length (bases), coverage	Circular 1: 16,000, 304×; Circular 2: 63,000, 264×; Coverage of the linears: 165×–248×, mean 174×	Linear, 152×	Circular : 6,609,466; Linear 1*: 11,000, 20×; Linear 2*: 2,000, 1×; Linear 3: 900, 18× * Linear contig 2 and 3 could be assembly artifacts based on either length or coverage
GC content average (%)	53	54.1	53
Bacterial strain deposition in a repository : accession no.	German Collection of Microorganisms and Cell Cultures DSM 113619, Belgian Coordinated Collections of Microorganisms LMG 32539, Culture Collection of Switzerland CCOS 2000	n.a.	n.a.
BioProject accession no.	PRJEB49674	PRJEB49674	PRJEB49674
BioSample accession no.	SAMEA14251711	SAMEA14509629	SAMEA14509628
Genome assembly accession no.	GCA_944800085	GCA_945605565	GCA_945318275
SRA accession no.	ERX9376482	ERX9376366	ERX9375765

^*a*^ Genome assemblies’ completeness was assessed with BUSCO [v4.0.6 (10), with option –auto-lineage-prok]. From the bacillales_odb10 database (creation date: 24 April 2019, number of species: 409, number of BUSCOs: 450), four BUSCO genes are missing in the *P. melissococcoides* assembly, one is fragmented, and six are complete but duplicated. These numbers are comparable to the reference strains *P. dendritiformis* LMG 21716 (missing: 2, fragmented: 0, duplicated: 6) and *P. thiaminolyticus* DMZ 7262 (missing: 2, fragmented: 1, duplicated: 15). Since the data of LMG 21716 were assembled into a single chromosome, six duplicated BUSCOs are presumably typical for *Paenibacillus* spp. Assemblies were annotated using PGAP (https://www.ncbi.nlm.nih.gov/genome/annotation_prok/, 2022-02-10.build5872), and the EMBLmyGFF3 tool (11) was used to convert the GFF3 annotation files into EMBL format to allow data submission to all three databases of the International Nucleotide Sequence Database Collaboration (INSDC).

^*b*^ n.a.; not applicable.

**Fig 1:**
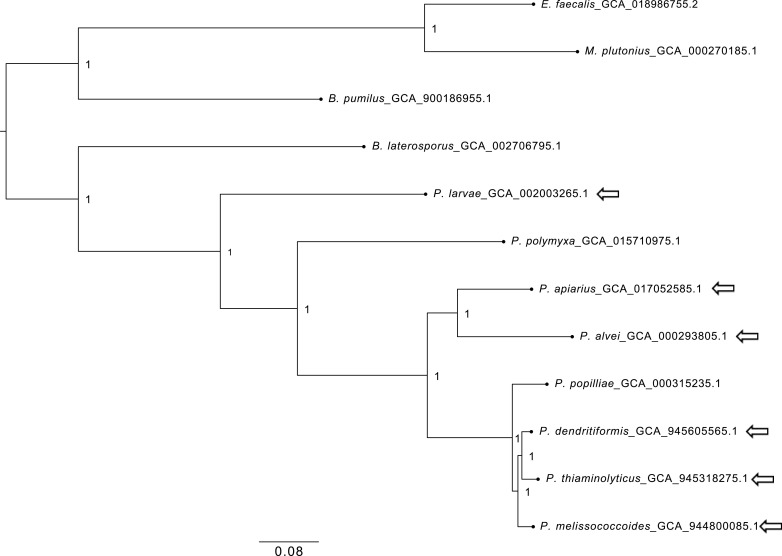
Species tree inferred from concatenated multiple sequence alignments of single-copy genes from *Paenibacillus* spp., *Bacillus* spp., and other bacteria that can be found in a honey bee colony. The tree was generated using Orthofinder v2.3.8 with option ‘-M msa’ (13 - 16) and rooted using STRIDE. STAG support values (15) are indicated at internal nodes, the species *M. plutonius*, *B. pumilus*, and *E. faecalis* were used as outgroup to root the tree. Arrows indicate *Paenibacillus* species associated with honey bees.

In a phylogenetic tree build using single-copy genes (Fig. 1), *P. melissococcoides* is located close to *P. dendritiformis* [ANI value 92.4%, calculated with fastANI v1.1 (12) using the genome assemblies] and *P. thiaminolyticus* (genome ANI value 91.1%) but distant from the known foulbrood pathogen *P. larvae*.

## Data Availability

See Table 1.
